# Viral Inhibition of Bacterial Phagocytosis by Human Macrophages: Redundant Role of CD36

**DOI:** 10.1371/journal.pone.0163889

**Published:** 2016-10-04

**Authors:** Grace E. Cooper, Zoe C. Pounce, Joshua C. Wallington, Leidy Y. Bastidas-Legarda, Ben Nicholas, Chiamaka Chidomere, Emily C. Robinson, Kirstin Martin, Anna S. Tocheva, Myron Christodoulides, Ratko Djukanovic, Tom M. A. Wilkinson, Karl J. Staples

**Affiliations:** 1 Clinical and Experimental Sciences, University of Southampton Faculty of Medicine, Sir Henry Wellcome Laboratories, Southampton General Hospital, Tremona Road, Southampton, SO16 6YD, United Kingdom; 2 Southampton NIHR Respiratory Biomedical Research Unit, Southampton General Hospital, Tremona Road, Southampton, SO16 6YD, United Kingdom; Nanyang Technological University, UNITED STATES

## Abstract

Macrophages are essential to maintaining lung homoeostasis and recent work has demonstrated that influenza-infected lung macrophages downregulate their expression of the scavenger receptor CD36. This receptor has also been shown to be involved in phagocytosis of *Streptococcus pneumoniae*, a primary agent associated with pneumonia secondary to viral infection. The aim of this study was to investigate the role of CD36 in the effects of viral infection on macrophage phagocytic function. Human monocyte-derived macrophages (MDM) were exposed to H3N2 X31 influenza virus, M37 respiratory syncytial virus (RSV) or UV-irradiated virus. No infection of MDM was seen upon exposure to UV-irradiated virus but incubation with live X31 or M37 resulted in significant levels of viral detection by flow cytometry or RT-PCR respectively. Infection resulted in significantly diminished uptake of *S*. *pneumoniae* by MDM and significantly decreased expression of CD36 at both the cell surface and mRNA level. Concurrently, there was a significant increase in IFNβ gene expression in response to infection and we observed a significant decrease in bacterial phagocytosis (p = 0.031) and CD36 gene expression (p = 0.031) by MDM cultured for 24 h in 50IU/ml IFNβ. Knockdown of CD36 by siRNA resulted in decreased phagocytosis, but this was mimicked by transfection reagent alone. When MDM were incubated with CD36 blocking antibodies no effect on phagocytic ability was observed. These data indicate that autologous IFNβ production by virally-infected cells can inhibit bacterial phagocytosis, but that decreased CD36 expression by these cells does not play a major role in this functional deficiency.

## Introduction

Respiratory infections are a leading cause of global morbidity and mortality. The especially high mortality rates in the 1918–19 influenza pandemic have been ascribed in part to secondary bacterial infection of the airway leading to pneumonia [1]. The major bacterial organism identified in these secondary bacterial pneumonias was *Streptococcus pneumoniae* [1]. This bacterial pathogen is also a feature of complications of seasonal influenza outbreaks, causing much of the morbidity and mortality associated with influenza on an annual basis [2].

Macrophages are key to the immune surveillance and defence of the human respiratory tract, being responsible for phagocytosis and clearance of infectious organisms [3]. Bacterial phagocytosis is a receptor-mediated event and numerous cell surface proteins have been implicated in the uptake of bacteria by macrophages including the Macrophage Receptor with Collagenous structure (MARCO) [4], CD36 [5] and CD206 [6]. There is increasing evidence that not only are macrophages targets of influenza infection [1], but also that the ability of macrophages from infected lungs to eliminate bacteria is compromised [4, 7–9]. A recent study utilising whole-genome microarrays has analysed the response of alveolar macrophages to influenza infection and has demonstrated a decrease in the gene expression of Dectin-1 (CLEC7A), Macrophage Scavenger Receptor (MSR)-1, CD206 (aka Mannose Receptor C 1) and CD36, which correlated with an inability of the macrophages to phagocytose zymosan [10].

CD36 is the prototypic class B scavenger receptor acting as a mediator of non-opsonic phagocytosis [11]. This receptor has been linked with both bacterial phagocytosis and proinflammatory signalling, as well as binding numerous ligands including oxidised low-density lipoproteins (oxLDL), thrombospondin-1 and long-chain fatty acids [5, 12–16]. CD36 has also been shown to have a role in *S*. *pneumoniae* phagocytosis in mice [17]. However, the mechanisms by which influenza infection affects CD36 expression and function of human macrophages has not been fully elucidated.

We have previously shown in an *ex vivo* lung model of influenza infection that influenza virus has tropism for both epithelial cells and macrophages [18]. In the present study, we have used a previously validated *in vitro* model of lung macrophages [19] to further investigate the role of CD36 in the effects of influenza infection on macrophage phagocytic function.

## Materials and Methods

### Ethics

The collection of lung tissue and blood was approved by and performed in accordance with the ethical standards of the South Central—Hampshire A Research Ethics Committee (LREC no: 13/SC/0416). Written informed consent was obtained from all participants.

### Monocyte isolation & differentiation

Monocytes were isolated from human peripheral blood mononuclear cells (PBMC) and differentiated into macrophages as previously described [19].

### Infection of MDMs

Influenza A virus strain X31 was supplied at a concentration of 4 x 10^7^ pfu/ml (a kind gift of 3VBiosciences). Inactivated virus (UVX31) was prepared by exposure of the X31 to an ultra-violet (UV) light source for 2 h. Macrophages were incubated for 2 h with no virus, 500 pfu (MDM) of X31 or UVX31. Cells were then washed and incubated for a further 22 h at 37°C, 5% CO_2_. Supernatants were harvested for cytokine analysis, HA shedding and LDH assays. Cells were collected using non-enzymatic cell dissociation solution (Sigma, Poole, UK) for immediate flow cytometry analysis or lysed and stored at -80°C for RNA analysis.

A similar method was used to infect MDM with Respiratory Syncytial Virus (RSV—strain M37—Meridian Life Science Inc, Memphis, USA). Inactivated RSV (UV-RSV) was prepared by exposure of the RSV to an ultra-violet (UV) light source for 45 min. 500 μl of stock RSV (3.5 x 10^6^ pfu) was diluted 1:1 in basal RPMI and a 1:10 dilution was made in each well and incubated for 2 h at 37°C. MDMs were then washed with basal RPMI and cultured in reduced serum (RS– 0.1%)-RPMI for a further 22 h.

### Flow cytometry analysis

Samples were resuspended in FACS buffer (PBS, 0.5% w/v BSA, 2 mM EDTA) containing 2 mg/ml human IgG before being incubated on ice in the dark for 30 min in the presence of fluorescently-labelled antibodies or isotype controls (all BD Biosciences, Oxford, UK). Intracellular staining for viral nucleoprotein (NP)-1, was performed using BD Cytofix/Cytoperm kit according to manufacturer’s instructions, and AlexaFluor 488 (AF488)-conjugated anti-NP1 antibody (HB-65, a kind gift of 3VBiosciences). Intracellular staining for RSV F protein was performed using BD Cytofix/Cytoperm kit according to manufacturer’s instructions, RSV-F protein antibody (Meridian Life Science Inc). After incubation on ice for 30 min, MDM were washed and incubated with AlexaFluor 488 anti-mouse antibody (Invitrogen, Paisley, UK). Flow cytometric analysis was performed on a FACSAria using FACSDiva software v5.0.3 (all BD).

### Phagocytosis experiments

MDM were infected with *Streptococcus pneumoniae* strain D39 serotype 2 at an MOI of 0.1 in RS-RPMI (without antibiotics) for 2 h in the presence or absence of 10 μg/ml CD36 blocking antibody or isotype control (Abcam, Cambridge, UK). MDMs were washed and incubated for 30 min in antibiotic-containing RS-RPMI to remove extracellular bacteria. Cells were then incubated in 1X Permwash (BD) for 20 min before vortexing and plating onto blood agar.

### RNA Isolation & RT-PCR

MDM were harvested in peqGOLD Trifast (Peqlab Lutterworth, UK)) and stored at -80°C prior to RNA extraction according to manufacturer’s instructions. RNA concentration was determined by NanoDrop 1000 (Thermo Scientific, Wilmington, USA) and 250 ng was mixed with 1X RT-buffer, 4mM dNTPs, 1X random primer, 50U Multiscribe Reverse Transcriptase and 20U RNase inhibitor (all from ThermoFisher, Basingstoke, UK) before thermocycling to generate cDNA. cDNA was diluted 1:10 and 1 μL mixed with 1X Taqman Universal MasterMix II, 1X Taqman Gene Expression Assay buffer and 0.25 μL of primers for RSV N gene, CD36, IFNβ and β2-microglobluin (β2M –all ThermoFisher), before undergoing qPCR. Gene expression was normalised to β2M and quantified using the ΔΔCT method.

### Supernatant analyses

IFNβ concentrations in culture supernatants were measured by ELISA according to the manufacturer’s instructions (MSD, Gaithersberg, USA). Culture supernatants were analysed for IL-10 by Luminex assay as per manufacturer’s instructions (Bio-Rad). LDH release was measured using CytoTox 96® Non-Radioactivity Cytotoxicity Assay according to the manufacturer’s instructions (Promega, Southampton, UK). Release of viral hemagglutinin was measured using a dot blot assay on nitrocellulose membrane detected using a rabbit polyclonal anti-influenza serum. The bound rabbit antibodies were detected using the Bio-Rad anti-Rabbit HRP detection system according to the manufacturer’s instructions.

### Transfection of MDM

HiPerfect immunocomplexes of 60 nM pooled CD36 siRNA sequences (Qiagen Crawley, UK) or scrambled RNA (AllStars Neg. siRNA AF488 Qiagen) were vortexed and incubated in the dark for 10 min at RT. MDM were incubated at 37°C in 100 μl RS-RPMI containing the immunocomplexes for 6 h before addition of 400 μl of RS-RPMI. MDM were then incubated for a further 18 h before further analysis. Transfection efficiency was calculated by analysis of fluorescently labelled scrambled RNA uptake by MDM using flow cytometry. The mean transfection efficiency was 96.33% (n = 5).

### Western blotting

After transfection MDMs were lysed in 150 mM Tris-HCl pH 8 containing 150 mM NaCl and 1% v/v Triton X-100. The lysates were agitated on ice for 30 min, before centrifugation at 12,000 g and supernatant harvest. Protein concentration was measured with the Pierce BCA Protein Assay Kit (ThermoFisher) according to manufacturer’s instructions and 30 μg of protein was loaded into a NuPage Novex 4–12% Bis-Tris Protein Gel (ThermoFisher). Electrophoresis was carried out at 200V for 1 h in NuPAGE MOPS SDS running buffer (ThermoFisher). Protein was then transferred to a PVDF membrane using the iBlot transfer system (Invitrogen) according to manufacturer’s instructions. After transfer the membranes were blocked for 1 h at RT in 5% non-fat dry milk (NFDM) in Tris-buffered saline (150mM NaCl, 50mM TrisHCl pH7.5) with 0.1%v/v Tween-20 (TBST). Membranes were then washed with TBST and incubated with either 1:1000 rabbit αCD36 (Abcam) or 1:50,000 α-β-actin-HRP (Abcam) for 1 h in NFDM-TBST at RT. Membranes stained for CD36 protein were washed again in TBST and incubated with 1:2000 goat α-rabbit-HRP (Sigma, Poole, UK) in NFDM-TBST for 1 h at RT. The membranes were then developed in the Novex ECL Chemiluminescent Substrate Reagent Kit (ThermoFisher) and imaged with the Chemidoc XRS imaging system (BioRad).

### Statistics

Statistical analyses were performed using a Wilcoxon’s signed rank test or paired Student’s t-test (GraphPad Prism v6, GraphPad Software Inc., San Diego, USA) as indicated. Results were considered significant if p<0.05.

For a full description of all the methods used please see Supporting Information S1 Methods

## Results

### Virally infected monocyte-derived macrophages (MDM) have a specific defect in bacterial phagocytosis

**Fig 1:**
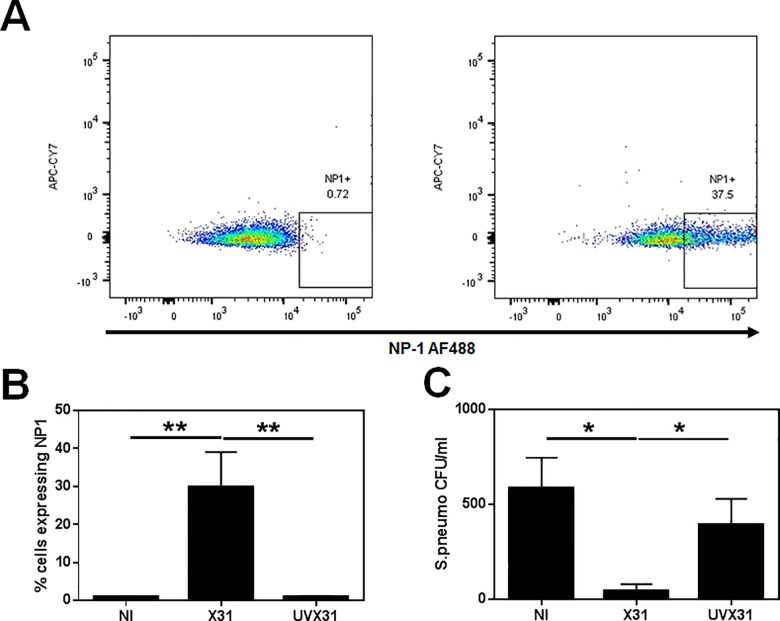
Effect of influenza infection on bacterial phagocytosis by MDM. MDM were differentiated in the presence of 2 ng/ml GM-CSF for 12 d prior to infection with H3N2 X31 influenza virus or a UV-irradiated aliquot of virus (UVX31) for 2 h. After washing, media was replaced and the cells incubated for a further 22 h before supernatants and cells were harvested for **(A) & (B)** influenza NP1 expression (% cells, n = 7) by flow cytometry. Phagocytosis of *S*. *pneumonia* was detected in **(C)** X31-infected (n = 5) MDM after a further 2 h incubation with live bacteria by culture. **(A)** Representative flow cytometry plot of MDM expressing influenza NP1. Data are expressed as means ±SE of n independent experiments and analysed using a Wilcoxon-signed rank test * p<0.05, ** p<0.01.

We infected MDM matured in the presence of 2 ng/ml GM-CSF for 12 d, as previously described [19]. After maturation, GM-CSF containing media was removed and replaced with RS RPMI. Cells were then incubated in the presence or absence (NT) of 500 pfu H3N2 X31 (X31) or UV-inactivated H3N2 X31 (UVX31) for 2 h. After a further 22 h, cells were collected and immediately analysed by flow cytometry. Using flow cytometry, 30% of MDM were NP-1+ after 22 h X31 infection, but no increase in NP-1 was detected in UVX31-exposed cells (Fig 1A and 1B).

**Fig 2:**
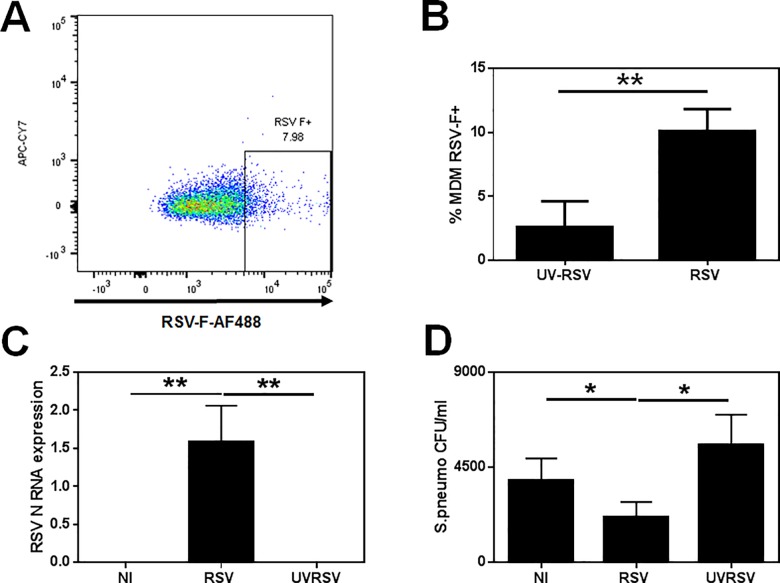
Effect of RSV infection on bacterial phagocytosis by MDM. MDM were differentiated in the presence of 2 ng/ml GM-CSF for 12 d prior to infection with M37 RSV or a UV-irradiated aliquot of virus (UV-RSV) for 2 h. After washing, media was replaced and the cells incubated for a further 22 h before supernatants and cells were harvested for **(A) & (B)** RSV-F protein expression (% cells, n = 10) by flow cytometry or **(C)** RSV-N gene expression by RT-PCR (n = 7). Phagocytosis of *S*. *pneumonia* was detected in RSV-infected (n = 6) MDM after a further 2 h incubation with live bacteria by culture. **(A)** Representative flow cytometry plot of MDM expressing RSV F-protein. The units for RSV-N gene expression are 2^ΔCT^. Data are expressed as means ±SE of n independent experiments and analysed using a Wilcoxon-signed rank test * p<0.05, ** p<0.01.

We next investigated if influenza had an effect on phagocytosis in our MDM model. MDM were infected as described above and after 24 h media was replaced and D39 *S*. *pneumoniae* was added for 2 h. After extensive washing in antibiotic containing media, cells were lysed and uptake of bacteria was assessed by culture. Phagocytosis of bacteria was significantly reduced in X31-infected MDMs by 92% (p = 0.0313), but not in UVX31 exposed cells (Fig 1C). Moreover this inhibitory effect on phagocytosis was not limited to influenza, as infecting the MDM with RSV (Fig 2A–2C) also significantly reduced uptake of bacteria by 45% (p = 0.0469—Fig 2D). However, influenza infection had no effect on the ability of MDMs to phagocytose latex microspheres (See S1 Fig).

### Effects of viral infection on CD36 expression by MDM

**Fig 3:**
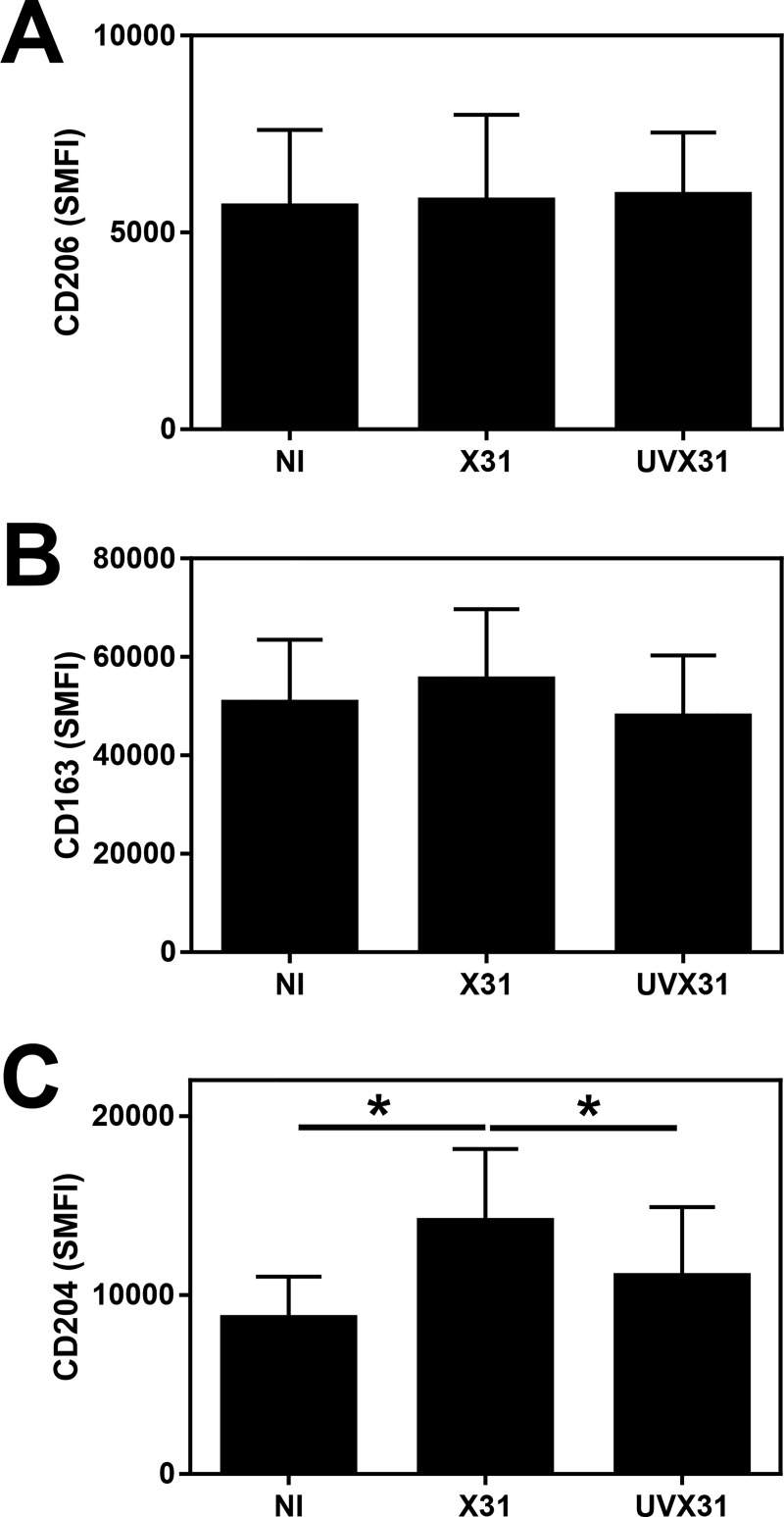
Effect of influenza infection on scavenger receptor expression by MDM. MDM were differentiated in the presence of 2 ng/ml GM-CSF for 12 d prior to infection with H3N2 X31 influenza virus or a UV-irradiated aliquot of virus (UVX31) for 2 h. After washing, media was replaced and the cells incubated for a further 22 h before supernatants and cells were harvested for cell surface **(A)** CD206 expression (n = 5), **(B)** CD163 expression (n = 5) or **(C)** CD204 expression by flow cytometry (n = 5). Data are expressed as means ±SE of n independent experiments and analysed using a Wilcoxon-signed rank test * p<0.05.

**Fig 4:**
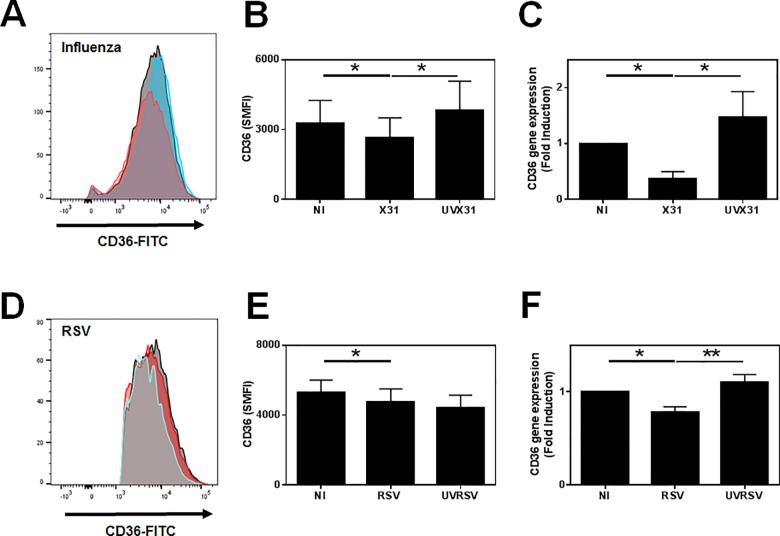
Effect of viral infection on CD36 expression by MDM. MDM were differentiated in the presence of 2 ng/ml GM-CSF for 12 d prior to infection with **(A) & (B)** H3N2 X31 influenza virus or a UV-irradiated aliquot of virus (UVX31) or **(C) & (D)** M37 RSV or a UV-irradiated aliquot of virus (UV-RSV) for 2 h. After washing, media was replaced and the cells incubated for a further 22 h before supernatants and cells were harvested for **(B) & (E)** CD36 cell surface expression by flow cytometry (specific mean fluorescence intensity–SMFI n = 6) by flow cytometry or **(C) & (F)** CD36 gene expression by RT-PCR (X31 n = 6, RSV n = 7). PCR data were normalised to β2MG and are expressed as mean fold induction over the non-infected (NI) sample ± SEM. Representative flow cytometry histograms of cell surface CD36 expression in response to **(A)** influenza infection and **(D)** RSV infection are shown. Grey = NI, Blue = UV virus, Red = Live virus. Cell surface data are expressed as means ±SE of n independent experiments and analysed using a Wilcoxon-signed rank test * p<0.05, ** p<0.01.

Since bacterial phagocytosis is a receptor mediated event, we analysed the expression of a range of receptors known to be involved in phagocytosis by multichromatic flow cytometry. We observed no significant difference in the surface expression of CD163 or CD206 but expression of the scavenger receptor A (CD204) was increased in response to influenza infection (Fig 3). In contrast, we observed a small but significant decrease in surface CD36 expression (p = 0.0469) on the surface of infected cells but not cells exposed to UVX31 (Fig 4B). This downregulation in CD36 surface expression was more pronounced at the level of mRNA expression, with X31 infected cells expressing less than half the amount (mean 0.37 ± S.E. 0.13) of CD36 steady state mRNA after 24 h (Fig 4C). A similar decrease in CD36 mRNA was also observed in RSV infected MDM, although not quite to the same extent (mean 0.78 ± SE 0.06—Fig 4F), but both live and UV-inactivated RSV reduced surface expression of this receptor (Fig 4E).

### Blockade of viral replication reverses downregulation of CD36

**Fig 5:**
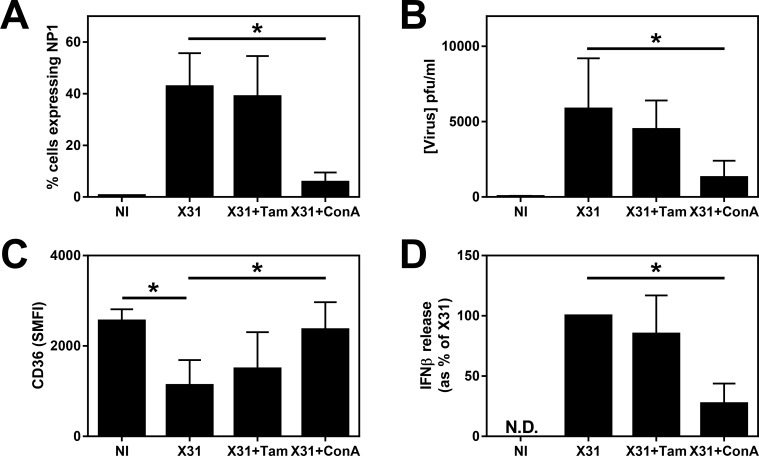
Effects of influenza infection on MDM cytokine expression. MDM were differentiated in the presence of 2 ng/ml GM-CSF for 12 d prior to infection with 500 pfu of H3N2 X31 influenza virus or a UV-irradiated aliquot of virus (UVX31) for 2 h in the presence or absence of oseltamivir phosphate (Tam– 10 μM) or concanamycin A (ConA– 10 nM). After washing, media containing inhibitors was replaced and the cells incubated for a further 22 h before supernatants and cells were harvested for **(A)** Viral NP1 expression (% cells, n = 6) by flow cytometry, **(B)** release of hemagglutinin into supernatants (n = 6), **(C)** CD36 cell surface expression by flow cytometry (SMFI–n = 6) and **(D)** IFNβ release by MSD are expressed as percentage of X31-infection (n = 6). Data are expressed as means ±SE of n independent experiments. Data analysed using a Wilcoxon-signed rank test. * p<0.05.

To ascertain the stage during the influenza life cycle that was essential to the down regulation of CD36, MDM were pretreated for 1 h with oseltamivir phosphate (10 μM—Tam) and concanamycin A (10 nM—ConA) before addition of X31 for a further 2 h and these compounds were added back to the media after the virus had been removed. Oseltamivir is a neuraminidase inhibitor that prevents viral shedding [20], but this compound could not reverse the decreased expression of CD36 on X31-infected MDM (Fig 5B). ConA inhibits viral replication by inhibition of endosomal acidification [21] and this compound significantly inhibited NP-1 expression (Fig 5A, p = 0.016) and prevented the downregulation of CD36 by infected MDM (p = 0.047—Fig 5C), suggesting replication of viral genes is essential in this process. Furthermore, we observed that in contrast to oseltamivir, ConA also significantly inhibited IFNβ release by X31-infected MDM (p = 0.031—Fig 5D).

### Effects of autologous cytokines released in response to viral infection

**Fig 6:**
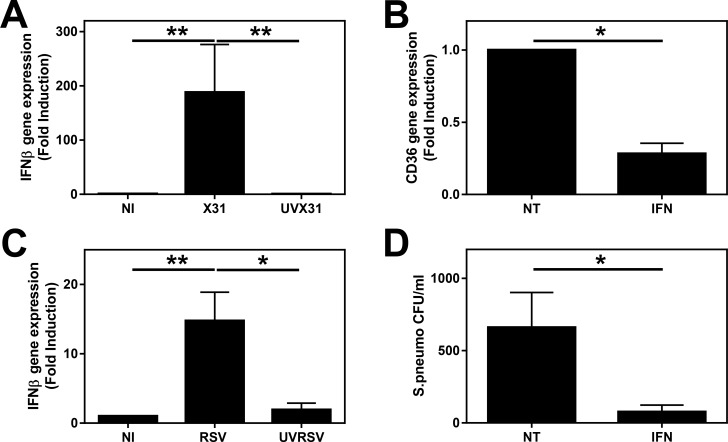
Virus-induced expression of IFNβ and effect of IFNβ on bacterial phagocytosis. MDM were differentiated in the presence of 2 ng/ml GM-CSF for 12 d prior to infection with **(A)** H3N2 X31 influenza virus or a UV-irradiated aliquot of virus (UVX31) or **(C)** M37 RSV or a UV-irradiated aliquot of virus (UV-RSV) for 2 h. After washing, media was replaced and the cells incubated for a further 22 h before supernatants and cells were harvested for IFNβ gene expression by RT-PCR (X31 n = 9, RSV n = 7). MDM were differentiated for 12 d as above before treatment without (NT) or with 50 IU/ml IFNβ for 24 h and **(B)** cells harvested for CD36 gene expression by RT-PCR (n = 5) or **(D)** phagocytosis of *S*. *pneumonia* was detected after a further 2 h incubation with live bacteria by culture (n = 5). PCR data were normalised to β2MG and are expressed as mean fold induction over the non-infected (NI) sample ± SEM. Data are expressed as means ±SE of n independent experiments and analysed using a Wilcoxon-signed rank test * p<0.05, ** p<0.01.

As the downregulation of CD36 by MDM was observed on the whole population of infected cells this suggests that this downregulation may be driven by autologous soluble factors. The previous data using ConA (Fig 5D) implicated IFNβ in the influenza-induced decrease of CD36. We therefore investigated whether IFNβ alone could cause a reduction in CD36 mRNA expression as well as bacterial phagocytosis in our model system. In agreement with our previous report [19], both influenza and RSV induced IFNβ gene expression from MDM (Fig 6A and 6C). When we incubated the MDM in 50 IU/ml IFNβ for 24 h we observed significant inhibition of CD36 mRNA expression by MDM (p = 0.031—Fig 6B). This dose of IFNβ significantly reduced the ability of MDM to phagocytose *S*. *pneumoniae* (p = 0.031—Fig 6D).

Previous work has also suggested a role for the anti-inflammatory cytokine, IL-10, in modulating macrophage function after influenza infection [22]. However, there was a trend towards decreased expression of IL-10 mRNA after 24 h infection by X31-infected MDM (See S2 Fig). Correspondingly, there was also no statistically significant increase of IL-10 release into the supernatants of infected MDMs (See S2 Fig).

### CD36 blockade is dispensable for bacterial phagocytosis

**Fig 7:**
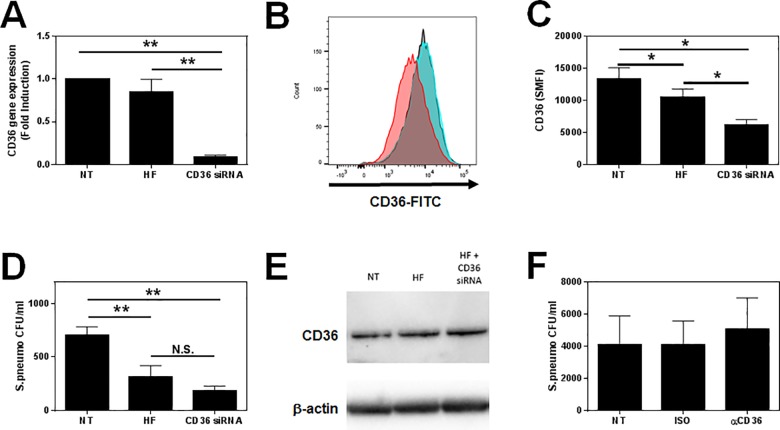
Role of CD36 in bacterial phagocytosis by MDM. **(A)-(E)** MDM were differentiated in the presence of 2 ng/ml GM-CSF for 12 d before transfection with non-specific siRNA or siRNA specific for CD36 for 24 h before cells were harvested for analysis of (**A)** CD36 mRNA expression by RT-PCR (n = 4) and **(B) & (C)** CD36 surface expression by flow cytometry (n = 6) or **(D)** phagocytosis of *S*. *pneumonia* was detected after a further 2 h incubation with live bacteria by culture (n = 4). **(E)** Total cellular expression of CD36 was assessed by western blot 24 h after transfection. β-actin is used as a loading control. Blot representative of 3 independent experiments. **(F)** MDM were differentiated for 12 d as above before phagocytosis of *S*. *pneumonia* was detected in the presence or absence of 10 μg/ml αCD36 blocking antibody or isotype control (ISO) after a further 2 h incubation with live bacteria by culture (n = 5). PCR data were normalised to β2MG and are expressed as mean fold induction over the not treated (NT) sample ± SEM of n independent experiments. **B)** Representative flow cytometry histograms of CD36 surface expression in response to transfection are shown. Grey = NT, Blue = HiPerfect (HF), Red = CD36 siRNA. Flow cytometry and phagocytosis data are expressed as means ±SE of n independent experiments. **(A) & (C)** Data analysed using a paired t-test ** p<0.01. **(B) & (D)** Data analysed using a Wilcoxon-signed rank test * p<0.05.

To investigate the role of CD36 on bacterial phagocytosis, siRNA was used to knockdown CD36 receptor expression by MDM. Specific siRNA targeting of CD36 caused a 92% reduction in CD36 mRNA (Fig 7A) with a 64.3% decrease at the cell surface, as measured by flow cytometry (Fig 7C). The agent used to deliver RNA, HiPerfect, was also shown to cause a small (19%), but significant decrease in CD36 protein expression compared to the non-transfected control. A correspondingly small, but not significant decrease in CD36 mRNA transcripts was also observed with empty HiPerfect.

Having demonstrated successful CD36 knockdown using this approach, *S*. *pneumoniae* uptake was assessed in CD36 siRNA transfected MDM (Fig 7D). Interestingly, a reduced uptake of *S*. *pneumoniae* was observed for cells transfected with empty HiPerfect, as well as those with CD36 siRNA. MDM transfected with RNA showed the greatest decrease in *S*. *pneumoniae* uptake. These results suggest that the transfection process itself affected the phagocytic ability of macrophages.

Since the HiPerfect reagent reduced CD36 surface expression (Fig 7C), these data could suggest that CD36 plays a pivotal role in the phagocytosis of *S*. *pneumoniae* by MDM. In an attempt to confirm this, we performed western blotting on transfected MDM (Fig 7E). In contrast to the surface expression data, we observed no effect of either the HiPerfect alone or the CD36 siRNA on total cellular expression of this protein, suggesting the surface loss of this protein in response to transfection may be due to CD36 internalisation. To further investigate the role of CD36 in *S*. *pneumoniae* phagocytosis we incubated the MDM in the presence of an antibody that blocks the CD36 receptor for 30 min prior to exposure to the bacteria. Blocking the CD36 receptor in this manner had no effect on the phagocytic ability of the MDM (Fig 7F).

## Discussion

This study has demonstrated that respiratory viral infection of macrophages inhibits the ability of these cells to phagocytose bacteria, and that this effect is likely driven by autologous release of IFNβ. In addition, although the expression of the phagocytic receptor CD36 was decreased by both viral infection and IFNβ, this receptor does not seem to be involved in the phagocytosis of bacteria by human macrophages as inhibition of CD36 interaction with bacteria through the use of blocking antibody had no effect on the uptake of bacteria by MDM.

Influenza infection of the airway has long been known to affect macrophage phagocytosis and bacterial killing [23], but the question as to whether this was mediated by exogenous factors or by infection of the macrophages themselves has remained open. Early studies demonstrated that virus-induced suppression of antibacterial defences was mediated by lymphocytes [24]. Subsequent studies suggested that it is the cytokines released by lymphocytes, such as IFNγ and IL-10, that mediate the suppressive effects of the virus [4, 7, 22, 25]. However, influenza can infect and replicate in human alveolar macrophages [26] and we have previously demonstrated that macrophages are a target of influenza infection in a human lung explant model [19]. The observed effects of virus on phagocytic ability are unlikely to be a result of virus-induced cell death as we have previously demonstrated that the dose of virus used in our MDM model does not cause increased cell death [19]. Furthermore, macrophages were shown to be relatively resistant to influenza-induced cell death [27]. Similarly to this previous study we have also observed no decrease in the ability of influenza-infected MDMs to take up fluorescent microspheres [27]. In contrast, the ability of MDMs to phagocytose live *S*. *pneumoniae* was significantly reduced, suggesting different mechanisms for the uptake of latex beads and bacteria.

Previous murine studies have demonstrated a role for both IL-10 and IFNγ in this virus-induced defect of phagocytosis [4, 22], yet we observed no expression of IFNγ mRNA or release in X31-infected MDM [19] and a trend towards a virally-induced decrease in IL-10 mRNA expression. These results suggest that, in contrast to murine models of influenza infection, neither IFNγ nor IL-10 are playing a role in the decreased bacterial phagocytosis. However, we did observe a possible role for virus-induced IFNβ release as a mediator of defective phagocytosis. The effects of type I IFN on macrophage phagocytosis are somewhat understudied in the literature. Stimulation of type I IFNs by the TLR3 agonist PolyI:C has been shown to increase pneumococcal nasopharyngeal colonisation in a murine model [8]. More recently in line with our data, IFNβ was also demonstrated to decrease the ability of human MDM to phagocytose the fungus, *Candida albicans* [28]. Intriguingly, IFNβ was previously reported to increase the ability of macrophages to phagocytose apoptotic cells [29]. These observations also have potential implications for the use of IFNβ in the prevention of virus-induced exacerbations of asthma [30], a disease already associated with decreased phagocytic function of macrophages [31].

Our data demonstrating influenza and RSV induced decreases in CD36 expression are in line with a recent report of gene expression changes in alveolar macrophages in response to influenza infection [10]. In addition, we are the first to report that IFNβ can also cause downregulation of this receptor. In contrast, our data regarding the functional relevance of this CD36 downregulation on bacterial phagocytosis are not so clear cut. Using siRNA that specifically targets CD36 we have demonstrated that we can reduce CD36 mRNA, cell surface expression and bacterial phagocytosis. Surprisingly, the siRNA transfection reagent alone induced a small, but significant, decrease in CD36 surface expression and subsequent reduction in bacterial phagocytosis. As CD36 is known to be involved in recognition and uptake of lipids as well as bacteria [11, 17] this could indicate that the HiPerfect, which is a mixture of cationic and neutral lipids, may inhibit bacterial phagocytosis by acting as a CD36 blocking agent. However, we observed no effect of the CD36 blocking antibody on bacterial phagocytosis, suggesting this postulation is unlikely. Furthermore, we observed no effect of transfection on total cellular expression of CD36 suggesting that this change at the cell surface may be mediated by internalisation of the protein. A further possibility for this effect of HiPerfect is suggested by the role of CD36 in the phagocytosis of apoptotic cells [32, 33]. In this case, HiPerfect may be mimicking apoptotic cells which may lead to similar anti-inflammatory and tolerogenic pathways being activated [34]. Further work is required to support this speculation.

The question remains is the virus-induced decrease in bacterial phagocytosis mediated by decreased scavenger receptor expression? The microarray data from influenza-infected alveolar macrophages published by Wang et al (2012), demonstrated that Dectin-1, CD204, CD206 and CD36 gene expression was decreased by infection [10]. Our data suggest no role for CD36, CD204 or CD206 in decreased bacterial uptake in response to viral infection. Dectin-1 is a C-type lectin that recognises fungal-derived glucans [10] and is therefore unlikely to be involved in the uptake of bacteria. Intriguingly, there is a defect in the ability of macrophages derived from patients with chronic obstructive pulmonary disease (COPD) to phagocytose bacteria that was not associated with any changes in a similar panel of scavenger receptors [35]. Thus, the decrease in phagocytic ability in response to viral infection and in COPD may be receptor independent. Further investigation into the mechanisms and pathways involved in decreased bacterial phagocytosis are therefore required.

In summary, we have shown that whilst viral infection and IFNβ treatment of macrophages downregulates both phagocytic ability and CD36 expression, the downregulation of CD36 appears to be uncoupled from bacterial phagocytosis. Instead, downregulation of this molecule by virus-infected MDM may reflect an attempt by the macrophage to return to homeostasis by reducing inflammatory potential since CD36 can mediate an inflammatory cascade [5]. The data reported herein also have implications for the use of lipid-containing transfection reagents for the study of pathways involved in bacterial phagocytosis.

## Supporting Information

S1 Fig Viability and cell surface marker expression of infected MDM. MDM were infected with X31 virus or UVX31 for 24 h before incubating with 0.4 μM YG latex microspheres at a ratio of 2.5 microspheres:1 cell. Phagocytosis of fluorescent beads was measured by flow cytometry. Data are expressed as means ± SE of 5 independent experiments and analysed using a Wilcoxon-signed rank test.
(PPTX)

S2 Fig Infection of MDM by influenza. MDM were differentiated in the presence of 2 ng/ml GM-CSF for 12 d prior to infection with H3N2 X31 influenza virus or a UV-irradiated aliquot of virus (UVX31) for 2 h. After washing, media was replaced and the cells incubated for a further 22 h before supernatants and cells were harvested for **A)** IL-10 gene expression by RT-PCR (n = 4) or **B)** IL-10 release (n = 6) by Luminex ELISA analysis of culture supernatants. PCR data were normalised to β2MG and are expressed as mean fold induction over the non-infected (NI) sample ± SEM. Data are expressed as means ±SE of n independent experiments and analysed using a Wilcoxon-signed rank test.
(PPTX)

S1 Methods A full description of all the methods used to generate the data contained within this paper. (DOC)
