# Supplementary figures and images for: Viral Inhibition of Bacterial Phagocytosis by Human Macrophages: Redundant Role of CD36
Source: PLoS One. 2016 Oct 4;11(10):e0163889. doi: 10.1371/journal.pone.0163889 (PMC5049764; doi:10.1371/journal.pone.0163889)

## Slide 1
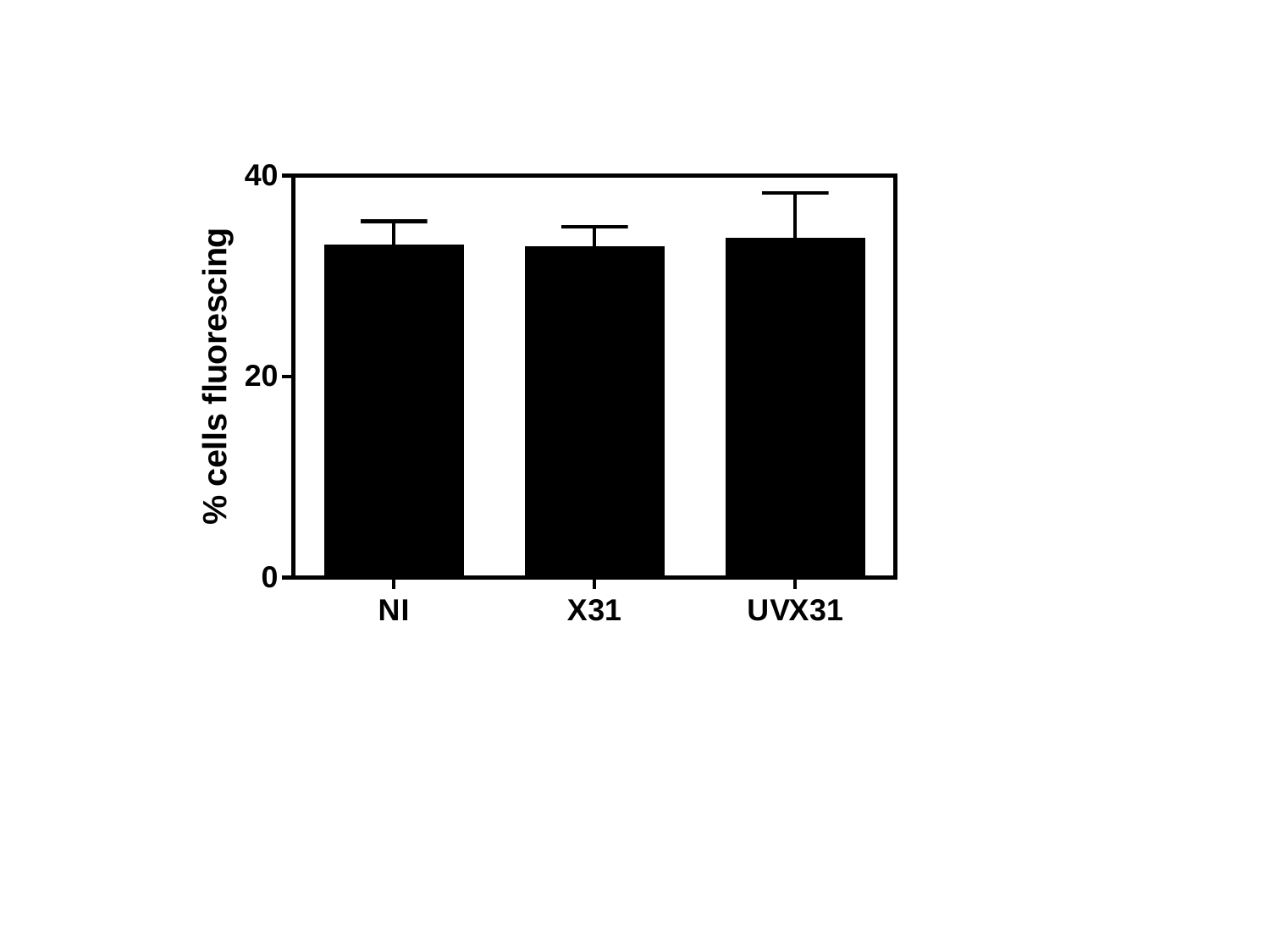

Supplement: S1 Fig — MDM were infected with X31 virus or UVX31 for 24 h before incubating with 0.4 μM YG latex microspheres at a ratio of 2.5 microspheres:1 cell. Phagocytosis of fluorescent beads was measured by flow cytometry. Data are expressed as means ± SE of 5 independent experiments and analysed using a Wilcoxon-signed rank test. (PPTX) [file pone.0163889.s001.pptx]

## Slide 1
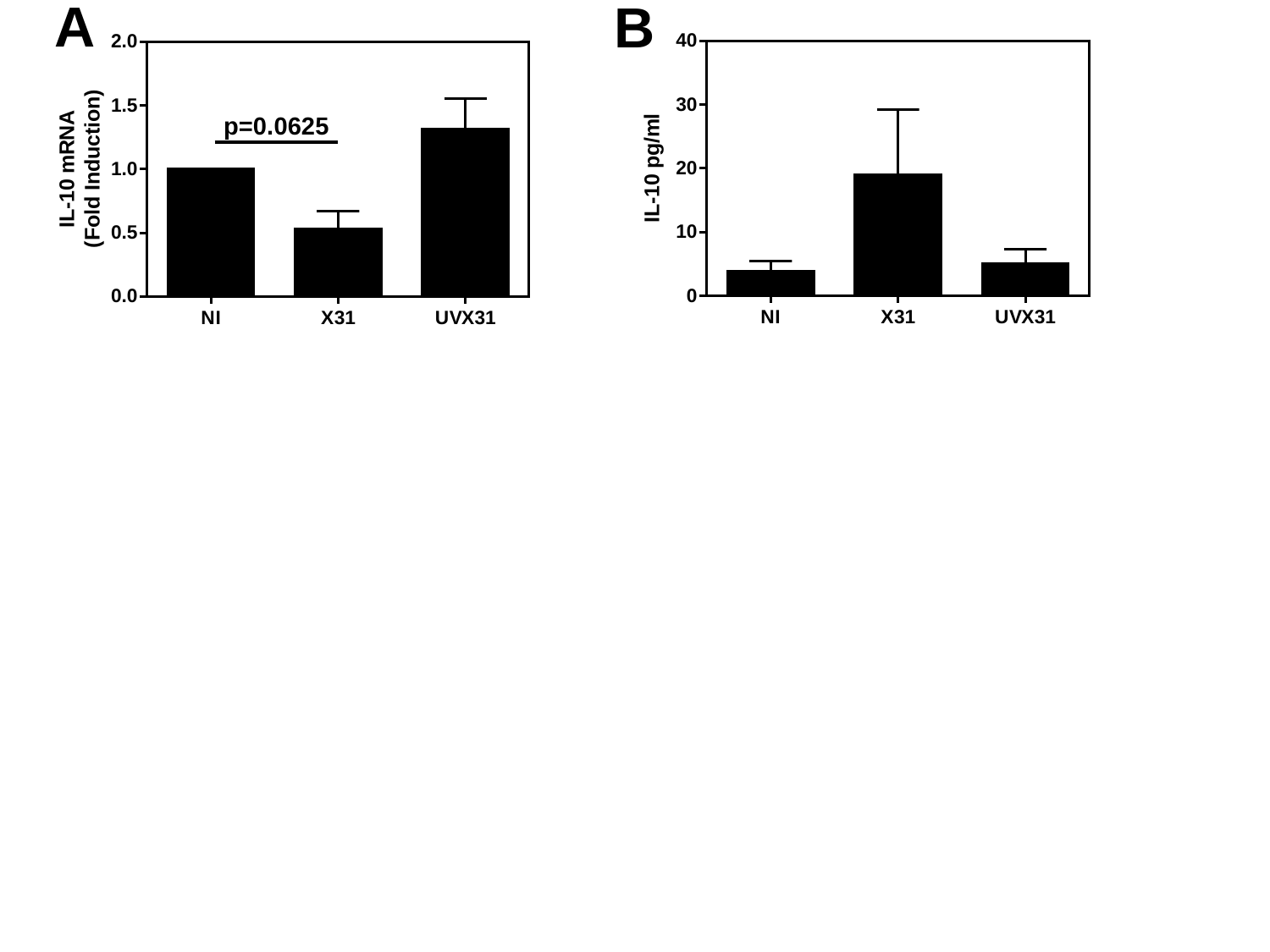

A
B

Supplement: S2 Fig — MDM were differentiated in the presence of 2 ng/ml GM-CSF for 12 d prior to infection with H3N2 X31 influenza virus or a UV-irradiated aliquot of virus (UVX31) for 2 h. After washing, media was replaced and the cells incubated for a further 22 h before supernatants and cells were harvested for A) IL-10 gene expression by RT-PCR (n = 4) or B) IL-10 release (n = 6) by Luminex ELISA analysis of culture supernatants. PCR data were normalised to β2MG and are expressed as mean fold induction over the non-infected (NI) sample ± SEM. Data are expressed as means ±SE of n independent experiments and analysed using a Wilcoxon-signed rank test. (PPTX) [file pone.0163889.s002.pptx]
