# Supplementary material for: Viral Inhibition of Bacterial Phagocytosis by Human Macrophages: Redundant Role of CD36
Source: PLoS One. 2016 Oct 4;11(10):e0163889. doi: 10.1371/journal.pone.0163889 (PMC5049764; doi:10.1371/journal.pone.0163889)
Supplement: S1 Methods — (DOC) [file pone.0163889.s003.doc]

**Supporting Information Methods for:**

**Viral inhibition of bacterial phagocytosis by human macrophages: role of CD36**

Grace E. Cooper^1^, Zoe C. Pounce^1^, Leidy Y. Bastidas-Legarda^1^, Joshua C. Wallington^1^, Ben Nicholas^1^, Emily C. Robinson^1^, Kirstin Martin^1^, Anna S. Tocheva^1^, Myron Christodoulides^1^, Ratko Djukanovic^1,2^, Tom M. A. Wilkinson^1,2^, Karl J. Staples^1^

**Methods**

**Monocyte Isolation & differentiation**

Human peripheral blood mononuclear cells (PBMC) were isolated from heparinised blood by centrifugation on Lymphoprep® (Axis-Shield, Oslo, Norway) or Ficoll-Paque (GE Healthcare). Monocytes were then isolated from the PBMC using CD14+ microbeads (Miltenyi-Biotec, Bisley, UK) according to the manufacturer’s instructions. Isolated monocytes were resuspended in complete RPMI supplemented with 2 ng/ml GM-CSF (R&D Systems, Abingdon, UK). MDM were then washed extensively with basal RPMI before addition of virus or rhIFNβ (National Institute for Biological Standards and Control, Potters Bar, UK) in RS-RPMI. MDM were harvested using non-enzymatic cell dissociation solution (Sigma, Poole, UK). Collection of samples for this part of the study was approved by the South Central - Hampshire A Research Ethics Committee (LREC no: 13/SC/0416).

**Viral infection of MDM**

Influenza A virus strain X31 was supplied at a concentration of 4 x 10^7^ pfu/ml (a kind gift of 3VBiosciences). Inactivated virus (UVX31) was prepared by exposure of the X31 to an ultra-violet (UV) light source for 2 h. Macrophages were incubated for 2 h with no virus, or 500 pfu (MDM) of X31 or UVX31. Supernatants were harvested (T-2), the cells washed three times, the final wash was harvested (T0) and fresh RS media was added to the MDMs. Cells were then washed and incubated for a further 22 h at 37°C, 5% CO_2_. After a further 22 h, supernatants were harvested (T22) for HA shedding and LDH assays and cells collected and immediately analysed by flow cytometry. For phenotypic characterisation of influenza infected macrophages, cells were removed from culture plates using a non-enzymatic cell dissociation solution (Sigma).

A similar method was used to infect MDM with Respiratory Syncytial Virus (RSV - strain M37 - Meridian Life Science Inc, Memphis, USA). Inactivated RSV (UV-RSV) was prepared by exposure of the RSV to an ultra-violet (UV) light source for 45 min. 500 µl of stock RSV (3.5 x 10^6^ pfu) was diluted 1:1 in basal RPMI; and a 1:10 dilution was made in each well and incubated for 2 h at 37°C. MDMs were then washed with basal RPMI and cultured in RS RPMI for a further 22 h.

**Flow cytometry analysis**

Samples were resuspended in FACS buffer (PBS, 0.5% w/v BSA, 2 mM EDTA) containing 2 mg/ml human IgG. Macrophages were incubated on ice in the dark for 30 min with the following antibodies: Fluorescein isothiocyanate (FITC)-conjugated anti-CD36, Allophycocyanin (APC)-conjugated anti-CD206 (both BD Biosciences), and PerCPCy-5.5-conjugated anti CD163 (Cambridge Bioscience, Cambridge, UK), or appropriate isotype controls. After washing, intracellular staining for viral nucleoprotein (NP)-1, was performed using BD Cytofix/Cytoperm kit according to manufacturer’s instructions, and AlexaFluor 488 (AF488)-conjugated anti-NP1 antibody (HB-65, a kind gift of 3VBiosciences).

Flow cytometric analysis was performed on a FACSAria using FACSDiva software v5.0.3 (all BD).

**Phagocytosis experiments**

MDM were infected with *Streptococcus pneumoniae* strain D39 serotype 2 at an MOI of 0.1 in RS-RPMI (without antibiotics) for 2 h in the presence or absence of 10 µg/ml CD36 blocking antibody or isotype control (Abcam, Cambridge, UK). MDMs were washed and incubated for 30 min in antibiotic-containing RS-RPMI to remove extracellular bacteria. Cells were then incubated in 1X Permwash (BD) in MIB for 20 min before vortexing and plating onto blood agar.

For microbead phagocytosis, MDM were incubated with YG latex microspheres (Polysciences Europe GmbH, Eppleheim, Germany) at a ratio of 2.5 microspheres per cell. MDM were then washed five times with DPBS before cells were harvested using non-enzymatic cell dissociation solution (Sigma). Cells were then fixed in 4% Paraformaldehyde for 20 min on ice prior to washing and resuspending in FACS buffer. Phagocytosis of microspheres was assessed using flow cytometry. Initial experiments demonstrated no effect of addition of trypan blue to quench extracellular fluorescence and thus trypan blue was not used in these experiments.

**Modulation of CD36 using siRNA**

HiPerfect immunocomplexes of 60 nM pooled CD36 siRNA sequences (Qiagen Crawley, UK) or scrambled RNA (AllStars Neg. siRNA AF488 Qiagen) were vortexed and incubated in the dark for 10 min at RT. MDM were incubated at 37⁰C in 100 µl RS-RPMI containing the immunocomplexes for 6 h before addition of 400 μl of RS-RPMI. MDM were then incubated for a further 18 h before further analysis. Transfection efficiency was calculated by analysis of fluorescently labelled scrambled RNA uptake by MDM using flow cytometry. The mean transfection efficiency was 96.33% (n=5).

**RNA Isolation & RT-PCR**

MDM were harvested in peqGOLD Trifast (Peqlab Lutterworth, UK)) and stored at -80°C prior to RNA extraction according to manufacturer’s instructions. RNA concentration was determined by NanoDrop 1000 (Thermo Scientific, Wilmington, USA) and 250 ng was mixed with 1X RT-buffer, 4mM dNTPs, 1X random primer, 50U Multiscribe Reverse Transcriptase and 20U RNase inhibitor (all from Thermo Fisher, Basingstoke, UK) before thermocycling to generate cDNA. cDNA was diluted 1:10 and 1 μL mixed with 1X Taqman Universal MasterMix II, 1X Taqman Gene Expression Assay buffer and 0.25 μL of primers for RSV N protein, CD36, IFNβ and β2-microglobluin (β2M – all Thermo Fisher), before undergoing qPCR. Gene expression was normalised to β2M and quantified using the ΔΔCT method.

**Supernatant analyses**

IFNβ concentrations in culture supernatants were measured by ELISA according to the manufacturer’s instructions (MSD, Gaithersberg, USA). Culture supernatants were analysed by Luminex assay for IL-2, IL-4, IL-5, IL-6, IL-8, IL-10, IL-12p70, IL-13, TNFα, and IFNγ as per manufacturer’s instructions (Bio-Rad). LDH release was measured using CytoTox 96® Non-Radioactivity Cytotoxicity Assay according to the manufacturer’s instructions (Promega, Southampton, UK). Release of viral hemagglutinin was measured using a dot blot assay. Briefly, culture supernatants were diluted 1:5 in PBS and transferred to pre-wetted nitrocellulose membrane in a 96-well array format using BioRad biodot apparatus. Standard curves were generated using purified virus preparations diluted in culture medium. Membranes were then blocked in 5% (w/v) non-fat dried milk powder in PBS-0.05% (v/v) Tween 20 for 1 h at room temperature. Viral proteins were detected using a rabbit polyclonal anti-influenza serum diluted 1/5,000 in blocking solution) overnight at 4^o^C. The bound rabbit antibodies were detected using the Bio-Rad anti-Rabbit HRP detection system according to the manufacturer’s instructions, where the secondary antibody (goat anti-rabbit HRP conjugate) was diluted 1/10,000 in PBS-Tween and incubated with the membrane for 2 h. Chemiluminscence images were captured using a Versadoc imager, and dot array quantities (OD/mm^2^) were analysed using QuantityOne software (BioRad, Hercules, Ca, USA). Specific viral release into the culture medium was analysed from the standard curve using sample values (pfu/ml) from which the background (post-infection) virus concentrations had been subtracted.

**Statistics**

Statistical analyses were performed using a Wilcoxon’s signed-rank test or paired Student’s t-test as indicated (GraphPad Prism v6, GraphPad Software Inc., San Diego, USA). Results were considered significant if p<0.05.
